# Validating the Demand Control Support Questionnaire among white-collar employees in Switzerland and the United States

**DOI:** 10.1186/s12995-018-0188-7

**Published:** 2018-02-13

**Authors:** Daniel Mauss, Raphael M. Herr, Töres Theorell, Peter Angerer, Jian Li

**Affiliations:** 1 Mannheim Institute of Public Health, Social and Preventive Medicine, Medical Faculty Mannheim, Heidelberg University, Ludolf-Krehl-Str. 7-11, D-68167 Mannheim, Germany; 2 Institute for Stress Research, Stockholm University, Stockholm, Sweden; 3 Institute of Occupational, Social and Environmental Medicine, Centre for Health and Society, Faculty of Medicine, University of Düsseldorf, Düsseldorf, Germany

**Keywords:** Demand-control-support model, Depressive symptoms, Job strain, Psychosocial stress, Validation

## Abstract

**Background:**

The Demand Control Support Questionnaire (DCSQ) is an established self-reported tool to measure a stressful work environment. Validated German and English versions are however currently missing. The aim of this study was therefore to evaluate the psychometric properties of German and English versions of the DCSQ among white-collar employees in Switzerland and the US.

**Methods:**

This cross-sectional study was carried out on 499 employees in Switzerland and 411 in the US, respectively. The 17-item DCSQ with three scales assessed psychosocial stress at work (psychological demands, decision latitude, and social support at work). Depressive symptoms were measured by the 2-item Patient Health Questionnaire. Cronbach’s α and item-total correlations tested the scale reliability (internal consistency). Construct validity of the questionnaire was examined using exploratory factor analysis (EFA). Logistic regressions estimated associations of each scale and job strain with depressive symptoms (criterion validity).

**Results:**

In both samples, all DCSQ scales presented satisfactory internal consistency (Cronbach’s α ≥ 0.72; item-total correlations ≥ 0.33), and EFA showed the 17 items loading on three factors, which is in line with the theoretically assumed structure of the DCSQ construct. Moreover, all three scales as well as high job strain were significantly associated with depressive symptoms. The associations were stronger in the US sample.

**Conclusions:**

The German and the English versions of the DCSQ seem to be reliable and valid instruments to measure psychosocial stress based on the job demand-control-support model in the workplace of white-collar employees in Switzerland and the US.

## Background

The job demand-control model proposed by Karasek [1] is one of the best evaluated models of stress at work [2]. It has been associated with the onset of major diseases such as depression [3] and cardiovascular disease [4, 5]. Furthermore, a reduction of this work-related stress can improve work ability, well-being and productivity of employees [6–8]. The job demand-control model takes psychological demands (e.g. workload and time pressure) and decision latitude (also called job control including decision authority and skill discretion) into consideration. High job strain usually leads to negative bodily responses (stress) and is defined as a mismatch of high demands and low decision latitude, whereas low strain is theoretically defined as low job demands with high decision latitude; meanwhile active job and passive job refer to both high job demands and decision latitude or both low, respectively. In 1988, the additional component of social support by peers and supervisors was added to the model as a possible resource to buffer the impact of job strain on health [9].

To operationalize the job demand-control-support model, two questionnaires were developed. The job content questionnaire (JCQ), which contains of 49 items in five scales such as decision latitude, psychological demands and mental workload, social support, physical demands, and job insecurity [10], has been validated in various languages and job profiles. The other instrument, the Demand Control Support Questionnaire (DCSQ), is an economical 17-item short form of the JCQ [11]. It comprises three scales of psychological demands (work fast, work intensely, work effort, overtime work, and conflicting demands), decision latitude divided in skill discretion (learning new things, skill level, being creative, variety of work), and decision authority (how to do the work, what to do at work), and social support at work (pleasant atmosphere, spirit of unity, colleagues support, helpful colleagues, relationship with superiors, relationship with colleagues). Thus, the DCSQ does not assess physical demands and is therefore much shorter and not that time-consuming compared to the JCQ.

Although the DCSQ has been validated in various languages like Brazilian-Portuguese [12, 13], Swedish [14], Norwegian [15], Turkish [16], and Japanese [17], no validated German or English version exists so far. Therefore, the aim of this study was to investigate the psychometric properties of the German version as well as the English version of the DCSQ among white-collar employees from Switzerland and the US. Firstly, the internal consistency reliability and construct validity of the questionnaires and its scales were analyzed. Secondly, we selected depressive symptoms as a criterion to test its associations of DCSQ scales and the job strain categories in order for criterion validity. In the past years, substantial evidence has been gained to establish a causal relation between job strain measured by the demand-control model and depressive symptoms [3, 18]. For instance, a recent review and meta-analysis showed that decision latitude (158,251 subjects in 19 studies) and job strain (197,682 subject in 14 studies) exerted the largest effects: an odds ratio for job strain was 1.74 to develop depressive symptoms, while high decision latitude protected against depressive symptoms with an odds ratio of 0.73 [3].

## Methods

### Study sample

A voluntary online questionnaire was offered to 1944 white-collar employees (48.4% female, 40.2 years) of a large multi-national insurance company in either German (944 employees in Switzerland) or English (1000 employees in the US). Employees in Switzerland were based in Zurich where German is the main language. Employees in the US were based in Minnesota. The study sample included various jobs of in-house staff, but no call center and sales activities. In total, 970 employees (537 from Switzerland and 433 from the US) answered the questionnaire (overall response rate = 49.9%). Participants with missing values were excluded from further analysis. The final sample comprised of 910 employees presenting a full data set with 499 participants from Switzerland and 411 from the US.

### Procedure

The survey was conducted in December 2014 during official office hours, and participation was anonymous. Sociodemographic characteristics included age (≤ 34, 35-49, ≥ 50 years), gender, job position (employee, employee with management responsibility), and tenure (< 5, 5-9, ≥ 10 years). Managers (employees with management responsibility) were defined as having a leadership role. Informed consent was given by each participant. Data collection and study procedure was approved by the corporate data protection officers and by the Ethical Committee of the Bavarian State Chamber of Physicians in Munich as the study was designed there at the headquarters of the company.

### Measurements

#### Demand Control Support Questionnaire (DCSQ)

The 17-item DCSQ includes the three scales of psychological demands (five items), decision latitude (six items), and social support at work (six items) [14]. In our present study, all the 17 items were expressed as statements and the respondents were asked to report their levels of agreement or disagreement on a four-point Likert scale, with higher values indicating higher psychological demands (range 5–20), higher decision latitude (range 6–24), and higher social support at work (range 6–24). All scale scores were calculated by summing up the respective unweighted item scores after appropriate reverse scoring of item 4 (overtime work) and item 9 (variety of work). As an English translated but not validated version of the DCSQ items already existed, there was no translational process from the original Swedish version into English needed. After reviewing national databases on literature of prior use of a German version, one of the authors (P.A.) translated the English DCSQ version into German supported by an English-speaking German translator. This first version was also reviewed and modified by the other German-speaking authors separately to rule out translational problems. All versions were returned to P.A. and consolidated for a consensual version. Finally, this version was approved by Prof. Theorell. Furthermore, a median split of the scale values of the samples were used to calculate low and high levels of psychological demands and decision latitude, and four groups of low strain, active, passive, and high strain were categorized accordingly.

#### Patient Health Questionnaire (PHQ-2)

Depressive symptoms were measured by the two-item Patient Health Questionnaire [19] using a four-point Likert scale (from “0 = not at all” to “3 = nearly every day”). Unweighted sum scores ranging 0-6 were calculated with scores ≥ 3 indicating a high risk for depressive symptoms. Cronbach’s α was 0.75 for the Swiss sample and 0.85 for the US sample.

### Statistical analysis

Based on the quality criteria of evaluating health-related questionnaires [20], three established psychometric properties were tested in our study: internal consistency reliability, construct validity, and criterion validity.

#### Consistency reliability

Internal consistency reliability refers to the overall consistency of a measure meaning that similar results are produced if conditions are consistent [20]. Internal consistency was evaluated by Cronbach’s α and item-total correlations. Values > 0.7 for Cronbach’s α and > 0.3 for item-total correlation were considered to be acceptable [21].

#### Construct and criterion validity

The validity of a measurement tool is the degree to which the tool measures what it claims to measure, including construct validity (i.e., extent to which a theoretical construct is measured) and criterion validity (i.e., comparison of the tool with other measures or outcomes) [20]. Construct validity was assessed by exploratory factor analysis (EFA) using principal axis extraction and varimax rotation. A factor loading ≥ 0.35 was considered satisfactory [22]. All the 17 items of the DCSQ were included in the EFA. Criterion validity was tested using multivariate logistic regression analyses to assess associations between the three DCSQ scales and depressive symptoms, adjusting for age, gender, job position, and tenure as these variables are known to influence the association of stress with depressive symptoms [18, 23, 24]. Results are shown as standardized odds ratios (ORs), defined as ORs per standard deviation change, with 95% confidence intervals (CIs). Furthermore, multivariate logistic regression models explored associations of the four stress groups (i.e., low strain, active, passive, and high strain) with depressive symptoms in both countries. A *p*-value < .05 was considered as statistically significant. SAS 9.4 (SAS Institute Inc., North Carolina, US) was used for all statistical analyses.

## Results

### Study population

Characteristics of both study samples including age, gender, job position, and tenure as well as depressive symptoms were comparable (see Table 1).

**Table 1:** Characteristics of study subjects (*N* = 910), N (%)

Variables		Switzerland (*N* = 499)	United States (*N* = 411)
Age	≤ 34 years	175 (35.07%)	119 (28.95%)
	35-49 years	201 (40.28%)	178 (43.31%)
	≥ 50 years	123 (24.65%)	114 (27.74%)
Gender	Men	264 (52.91%)	171 (41.61%)
	Women	235 (47.09%)	240 (58.39%)
Job position	Manager	78 (15.63%)	96 (23.36%)
	Employee	421 (84.37%)	315 (76.64%)
Tenure	< 5 years	225 (45.09%)	133 (32.36%)
	5-9 years	90 (18.04%)	111 (27.01%)
	≥ 10 years	184 (36.87%)	167 (40.63%)
Depressive symptoms		63 (12.62%)	54 (13.14%)

### Consistency reliability

Mean values with standard deviations (SDs) of the three scales are presented in Table 2. Compared to the Swiss sample, demands and control were slightly higher whereas social support was slightly lower in the US sample. Cronbach’s α indicated satisfactory internal consistency for all three scales (psychological demands, decision latitude, and social support) of the German (0.72, 0.77, and 0.83, respectively) and the English version (0.78, 0.78, and 0.84, respectively). Values for item-total correlations varied between 0.33 and 0.69 for the German version, and between 0.38 and 0.73 for the English version indicating satisfactory internal consistency.

**Table 2:** Mean, standard deviation (SD), item-total correlation and Cronbach’s alpha coefficients of the scales of the DCSQ

Items	Switzerland			United States		
	Mean (SD)	Item-total correlations	Alpha of scale	Mean (SD)	Item-total correlations	Alpha of scale
Psychological demands (scale range: 5-20)	12.59 (2.44)		0.72	13.27 (2.43)		0.78
Item 1: work fast		0.59			0.58	
Item 2: work intensively		0.50			0.57	
Item 3: work effort		0.48			0.54	
Item 4: overtime work		0.51			0.55	
Item 5: conflicting demands		0.33			0.54	
Decision latitude (scale range: 6-24)	16.60 (3.00)		0.77	17.31 (2.84)		0.78
Item 6: learning new things		0.45			0.54	
Item 7: skill level		0.48			0.38	
Item 8: being creative		0.64			0.62	
Item 9: variety of work		0.45			0.49	
Item 10: how to do the work		0.57			0.60	
Item 11: what to do at work		0.50			0.57	
Social support at work (scale range: 6-24)	18.88 (2.82)		0.83	18.34 (2.68)		0.84
Item 12: pleasant atmosphere		0.62			0.52	
Item 13: spirit of unity		0.68			0.73	
Item 14: colleagues support		0.69			0.69	
Item 15: helpful colleagues		0.53			0.52	
Item 16: relationship with superiors		0.49			0.65	
Item 17: relationship with colleagues		0.66			0.59	

In addition, we observed significant differences according to gender (female employees had lower decision latitude, in the Swiss sample only), age (decision latitude increased with higher age in both samples), and position (managers had higher psychological demands and decision latitude in both samples), suggesting good discriminant validity (data not shown).

### Construct validity

Results of the explanatory factor analysis are presented in Table 3. In accordance with the criterion of Kaiser’s eigenvalue > 1 and the scree plot analysis, a three-factorial solution was applied. In both the Swiss and the US sample, the factor patterns were similar and the hypothesized factorial structure was well identified. All items of a respective scale were loaded onto one of the three factors with factor loadings between 0.38 and 0.77.

**Table 3:** Exploratory factor analysis of the 17-item DCSQ using principal axis extraction and varimax rotation

Scales	Items	Switzerland			United States		
		F1	F2	F3	F1	F2	F3
Psychological demands	Item 1: work fast			0.72		0.66	
	Item 2: work intensively			0.67		0.66	
	Item 3: work effort			0.51		0.59	
	Item 4: overtime work			0.60		0.61	
	Item 5: conflicting demands			0.38		0.60	
Decision latitude	Item 6: learning new things		0.47				0.54
	Item 7: skill level		0.55				0.45
	Item 8: being creative		0.71				0.67
	Item 9: variety of work		0.52				0.55
	Item 10: how to do the work		0.66				0.65
	Item 11: what to do at work		0.60				0.61
Social support at work	Item 12: pleasant atmosphere	0.68			0.44		
	Item 13: spirit of unity	0.72			0.70		
	Item 14: colleagues support	0.77			0.77		
	Item 15: helpful colleagues	0.57			0.48		
	Item 16: relationship with superiors	0.52			0.66		
	Item 17: relationship with colleagues	0.73			0.73		
Variance explained (%)		17.73	13.19	11.52	16.18	15.93	13.96

Only items with factor loading ≥ 0.35 are shown

### Criterion validity

In multivariate logistic regression analysis, for every SD increase in psychological demands a significantly (*p* < .001) elevated standardized OR of 1.86 for depressive symptoms was observed in the Swiss sample. For every SD increase in decision latitude and social support a decreased standardized OR of 0.52 and 0.40 respectively was observed for depressive symptoms. Notably, the magnitude of associations with depressive symptoms was relatively larger in the US sample (Table 4). Compared to low strain, high strain showed a standardized OR of 5.91 (*p* < .001) in Switzerland and 12.22 (*p* < .001) in the US after adjusting for age, gender, job position, and tenure.

**Table 4:** Associations of the scales of the DCSQ with depressive symptoms (standardized odds ratios (ORs) and 95% confidence intervals (CIs))

		Switzerland	United States
Psychological demands	Increase per SD	1.86 (1.40, 2.47) ***	2.20 (1.57, 3.08) ***
Decision latitude	Increase per SD	0.52 (0.39, 0.71) ***	0.41 (0.30, 0.57) ***
Social support at work	Increase per SD	0.40 (0.29, 0.54) ***	0.47 (0.34, 0.66) ***
Low strain		1.00	1.00
Active		2.58 (0.96, 6.96)	3.55 (1.06, 11.91) *
Passive		3.55 (1.33, 9.47) **	4.01 (1.19, 13.50) **
High strain		5.91 (2.28, 15.30) ***	12.22 (4.06, 36.83) ***

Multivariate logistic regression, * *p* < .05, ** *p* < .01, *** *p* < .001

Adjusted for age, gender, job position, and tenure

## Discussion

The aim of the present study was to validate the German and English versions of the DCSQ in white-collar employees in Switzerland and the US. Our results demonstrate a good internal consistency, construct validity, and criterion validity across both samples.

The properties of the German and English DCSQ versions are in line with findings from validation studies in other languages. Cronbach’s alpha coefficients explored in our samples are similar to DCSQ versions validated in Brazil [12, 13], Sweden [14], Norway [15], Turkey [16], and Japan [17]. As supported by these studies, social support showed the highest internal consistency in both samples of our study. However, our results indicated lower sum scores for psychological demands and decision latitude compared to other DCSQ validation studies [14, 17]. Regarding discriminant validity we observed significant differences according to age, gender, and position, which are consistent with previous studies, showing that women have lower decision latitude [25], decision latitude increases with age [26], and managers having higher psychological demands and decision latitude [27]. The DCSQ factorial patterns in the two samples from Switzerland and the US reflected well the theoretical structure and the three-factor solution of this questionnaire. Explanatory factor analysis in Norwegian workers [15] and Japanese nurses [17] confirmed this structure as well. On the other hand, a recent longitudinal study using confirmatory factor analysis found a four-factor structure, dividing decision latitude into the two subscales of skill discretion and decision authority [14]. We conducted supplementary EFA among the six items of the decision latitude scale, two distinct factorial loadings representing skill discretion and decision authority were also observed (data not shown).

Although internal consistency and construct validity were similar between the Swiss and the US sample, criterion validity is somewhat different. It seems like the associations of psychosocial stress in the workplace with depressive symptoms were stronger in the US sample than in the Swiss sample. It might be the case that protective labor and social policies modify the strength of these associations. A study assessing depressive symptoms among 14,236 older employees experiencing higher levels of psychosocial stress at work found higher risk estimation in the US (OR = 2.28) compared to Europe (OR = 1.97) and Japan (OR = 1.64) in cross-sectional data, while longitudinal analysis indicated somewhat smaller but still significant associations [28]. To explain these results, Lunau and colleagues longitudinally explored the impact of national labor and social policies on this association in European countries and the US. They used six macro indicators defined by the Organization for Economic Co-operation and Development (OECD) including active labor market policy expenditures, investments in rehabilitation services, participation of older employees in learning offerings, support for unemployed people, degree of union density, and income inequalities. Switzerland scored better for every one of these six indicators than the US with the Scandinavian countries having the best scores. It was suggested these macro indicators modified the effect of work-related stress on depressive symptoms across countries [29]. As for our present study, given the consideration that national labor and social policies in Switzerland are generally more protective than those in the US, the effect of work stress might be less health threatening if stress is experienced in a protective society. Interestingly, an active job which is defined as combination of high job demands with high decision latitude is usually assumed to be health-promoting [10]. In contradiction to that, in our sample an active job was associated with higher poor mental health compared to a low strain job, although this finding was not significant in the Swiss sample and slightly significant in the US sample. A similar result was described by Madsen in a recent meta-analysis [18].

### Limitations and strengths

Our study has some limitations to address. First, the analysis is based on cross-sectional data. That did not allow us to test for longitudinal stability and test-retest reliability. Second, we could not rule out the healthy worker effect, i.e., employees with reduced health status have been sick at home or had already left the company and therefore did not participate in this study. Third, the study sample was comprised of employees of a multi-national insurance company based in Switzerland and the US. Generalization to other industries in both countries as well as to other languages within Switzerland (French, Italian, Swiss-German, Rhaeto-Romanic) cannot be made. As Swiss people are usually very familiar with German, especially in Zurich, we do not think that using a German questionnaire may have biased the results due to any dialectical misunderstandings. Additionally, the fact that all variables had been measured by a self-rated questionnaire could lead to common method variance bias. Fourth, although the PHQ-2 is a validated instrument to screen depressive symptoms, it is based on two cardinal symptoms only (“little interest” and “feeling down”). Therefore, it must be distinct from clinically diagnosed depression [30]. Nevertheless, these limitations are balanced by several strengths. Compared to some of the other DCSQ validation studies [13, 16, 17] the sample size of our study was somewhat bigger providing good statistical power and allowing us to test the categorized job strain model (low strain, active job, passive job, high strain). Furthermore, our study is the first to validate the psychometric properties of the German and English versions of the DCSQ. This validation of the 17-item parsimonious and economical instrument would provide more option for studies on psychosocial working conditions and health outcomes in German and English speaking countries.

## Conclusions

Both the German and the English versions of the DCSQ seem to be reliable and valid instruments to measure psychosocial stress in the workplace, based on the job demand-control-support model, in white-collar employees in Switzerland and the US.

## Abbreviations

DCSQ: Demand control support questionnaire
EFA: Exploratory factor analysis
JCQ: Job content questionnaire
OECD: Organization for economic co-operation and development
OR: Odds ratio
PHQ-2: Patient health questionnaire
SD: Standard deviation
US: United States of America
